# A suspended graphene-based optical interferometric surface stress sensor for selective biomolecular detection†

**DOI:** 10.1039/c9na00788a

**Published:** 2020-03-19

**Authors:** Shin Kidane, Hayato Ishida, Kazuaki Sawada, Kazuhiro Takahashi

**Affiliations:** Toyohashi University of Technology Toyohashi Aichi 441-8580 Japan takahashi@ee.tut.ac.jp

## Abstract

Graphene-based sensors are of great interest in research due to their high specific surface area and high electron mobility that make them suitable for numerous advanced applications. In this paper, selective molecular detection using an antigen–antibody reaction on suspended graphene with a cavity-sealing structure was demonstrated. The suspended graphene sealed nanocavities in a pre-patterned Si substrate, which increased robustness and allowed the use of wet chemical processes for surface functionalization of the suspended graphene to achieve selective molecular binding. The selectivity was evaluated by nanomechanical deflection induced by molecular adsorption on the suspended graphene, resulting in spectral shifts in the optical interference between the suspended graphene and Si substrate. The chemically functionalized suspended graphene enables the analysis of intermolecular interactions and molecular kinetics by colorimetry using optical interference.

## Abstract 2

Chemically functionalized suspended graphene with a cavity-sealing structure selectively detects biomolecules using nanomechanical deflection associated with molecular adsorption.

## Graphical abstract

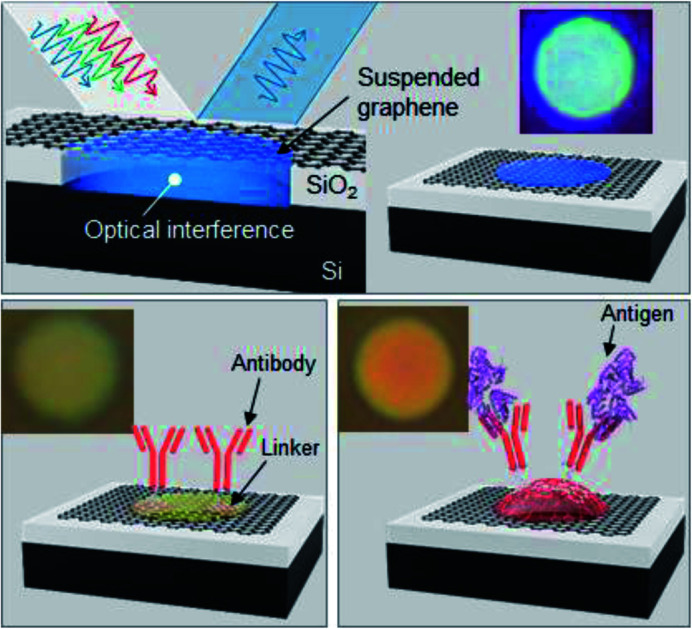

Various transducers, including those based on charge detection,^1–3^ resonant mass detection,^4–6^ and surface stress detection,^7–12^ have been studied as miniaturized sensors for detecting chemical substances^7^ and biomolecules such as DNA,^9^ RNA,^10^ proteins,^11^ and viruses.^12^ To improve the sensitivity of these sensors, graphene-based sensors are receiving considerable attention owing to their high specific surface area, ability to form ultrathin films, and high electron mobility.^13^ For example, a maximum electron mobility of 2 × 10^5^ cm^2^ V^−1^ s^−1^ was obtained when phonon scattering from the substrate was eliminated,^14^ resulting in a highly sensitive chemical sensor that detected resistance changes associated with adsorption of a target molecule. In addition, when suspended graphene is used as a resonant membrane, a mass sensor capable of detecting single molecules can be fabricated. In surface stress sensors, adsorbed molecules induce surface stress, resulting in a deflection of the suspended membrane. In particular, surface stress sensors can analyze the kinetics of molecules by transducing the interactions of adsorbed molecules into nanomechanical deflections.^15,16^ We previously developed a surface stress sensor based on optical interferometry to increase stress sensitivity using an optomechanical transducing technique.^17–19^

For chemical and biomedical sensing, the interface structure of the sensor surface is important. It has been reported that a single molecule of carbon dioxide was detected on suspended graphene.^20,21^ A resistance change of a suspended graphene channel caused by adsorption of a single molecule on the bilayer graphene bridge was demonstrated. However, this sensor was based on the physical adsorption of carbon dioxide, and selective detection in an environment with contaminants is impossible. Molecules cannot selectively bind to pristine graphene as it has no dangling bonds on its surface. As molecular selectivity is necessary for the application of suspended graphene as a sensor, the graphene surface needs to be functionalized. It has been reported that a specific protein was detected using an ion-sensitive field-effect transistor (ISFET)-based biosensor modified with an antibody fragment on a graphene channel fixed to a substrate.^22,23^ This chemical modification was carried out using a wet treatment, where graphene was immersed in a solution with dissolved receptor molecules. Note, however, that the detection area of the ISFET-based biosensor is restricted to 2–3 nm from the device surface due to the Debye length under physiological conditions, and therefore it is difficult to detect charged macromolecules.^24^ On the other hand, surface stress-based biosensors are unconstrained by the molecular size because they is based on the principle of detecting repulsive forces due to intermolecular interactions of adsorbed molecules as nanomechanical deflection,^25,26^ resulting in the detection of biomacromolecules such as proteins. However, chemical functionalization of the graphene bridge for selective molecular detection is difficult because the liquid trapped in the gap of the structure can easily fracture the suspended graphene by surface tension forces. To solve this problem, drum-type suspended graphene can prevent solution from entering the cavity, allowing wet treatments.

Deposition techniques for producing graphene include mechanical exfoliation,^27^ chemical vapor deposition (CVD) on a metal catalyst, such as copper^28^ or nickel,^29^ and epitaxial growth on silicon carbide.^30^ For large-area device fabrication, transfer techniques have been used, where CVD graphene was deposited on a Cu catalyst by thermal CVD, followed by removal of the Cu catalyst, and transferred to an arbitrary substrate.^31,32^ By transferring CVD graphene onto a Si substrate with pre-patterned cavities, the suspended graphene sealed the cavities and formed a drum-like structure.^33^ To obtain cavity sealing graphene with a drum structure, we proposed a low-pressure dry transfer technique.^34^ CVD graphene was transferred under vacuum to increase the real contact area between the substrate and transferred graphene in order to provide strong adhesion between the transferred CVD graphene and the substrate. The suspended graphene seals the cavities and provides sufficient robustness to allow wet chemical processing in order to perform molecular modification of the suspended graphene for selective molecular binding.

In this paper, we demonstrate selective molecular detection using an antigen–antibody reaction with such graphene drum sensors fabricated using our low-pressure dry transfer technique. Selective molecular detection was evaluated by the nanomechanical deflection induced by molecular adsorption on the suspended graphene, which resulted in a spectral shift in the optical interference between the suspended graphene and the Si substrate. The proposed technique can analyze intermolecular interactions by colorimetry using optical interference.

As for our previously reported low-pressure dry transfer technique, CVD graphene held by a temporary polymethyl methacrylate (PMMA) supporting sheet was transferred onto a Si substrate with pre-patterned nanocavities under vacuum (ESI Fig. S1†). By placing the graphene and SiO_2_ surfaces in contact under vacuum, the adhesion area increases due to deaeration of the gap between the two surfaces. By heating at a temperature higher than the glass transition temperature of PMMA, the fluidity of PMMA increases, resulting in a further increase in the real contact area. Finally, the PMMA layer is removed to leave a freestanding drum-like suspended graphene layer over the nano/microcavities, as shown in Fig. 1(a).

**Fig. 1:**
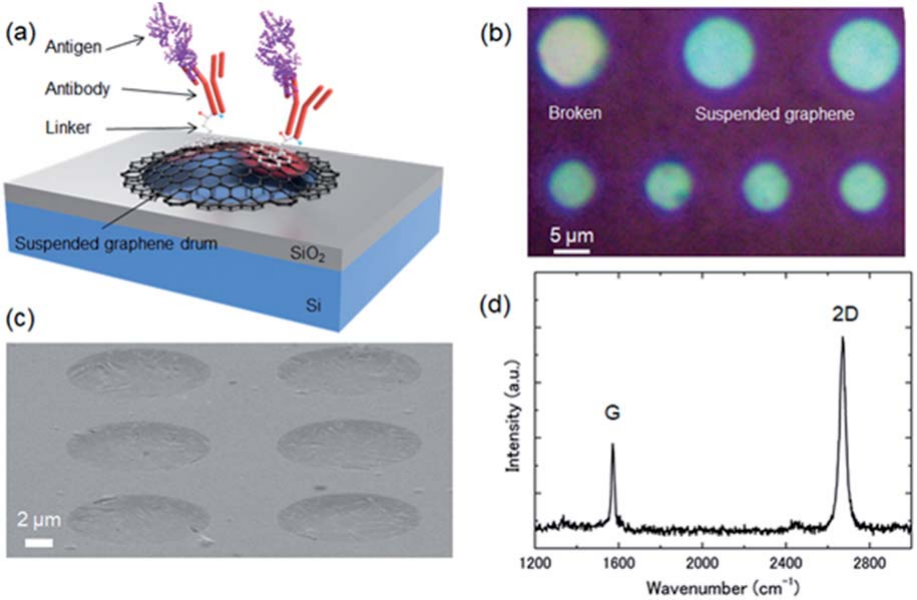
(a) Schematic of a suspended graphene-based optical interferometric surface stress biosensor. (b) Optical micrograph and (c) SEM image of the graphene drum with sealed cavities fabricated by the low-pressure dry-transfer technique. (d) Typical Raman spectrum of the suspended graphene drum with 2D/G and D/G peak ratios of 2.2 and 0.08, respectively.

Fig. 1(b) and (c) show optical microscopy and scanning electron microscopy (SEM) images, respectively, of a representative cavity-sealed suspended graphene sample. Although some cracks in the graphene were observed, we confirmed that the graphene was suspended above the cavity. The diameter of each suspended area was 6–10 μm. We previously reported cavities sealed under vacuum using the suspended graphene with diameters of up to 4.5 μm.^34^ Therefore, it is considered that graphene of this diameter contains some defects in the suspended area. It has been reported that the hydrophobic surface of a biosensor device prevented flooding of the gap when molecular modification was performed in solution.^35^ Even with a suspended membrane containing cracks of several microns, it seemed that the hydrophobic graphene did not allow permeation of liquid into the cavity. To evaluate the crystal quality, the Raman spectra of the suspended graphene were measured using a laser Raman spectrometer (JASCO, NSR-7100). Fig. 1(d) shows a typical Raman spectrum of a suspended graphene drum. The obtained 2D/G and D/G peak ratios were 2.2 and 0.08, respectively, which confirmed that the suspended graphene was a single layer.

Surface stress measurements were used to evaluate selective molecular detection on the suspended graphene. The surface stress sensor measures a static deflection of a suspended membrane caused by the electrostatic repulsive force from adsorbed biomolecules.^25,26^ With this sensing method, we obtained real-time responses of molecular binding in a liquid environment. Unlike a FET-type biosensor whose measurement distance from the sensor surface is restricted by the Debye length, the surface stress sensor can detect the response from biomacromolecules adsorbed far from the sensor surface, which allows the use of macromolecules, such as antibodies, as receptors. The sensitivity of the surface stress sensor is inversely proportional to Young's modulus and the square of the film thickness.^36^ Therefore, the use of 2D materials with an atomic-scale thickness can increase the static deflection and hence, the sensitivity of measuring the surface stress. By using a suspended graphene membrane, the sensitivity is expected to increase compared to that of conventional surface stress sensors, which have a typical thickness of 100–1000 nm, even though the Young's modulus of graphene is up to 1 TPa.^37^ In addition, the suspended graphene is a highly sensitive chemical and a biosensor with a small footprint, while conventional MEMS-based surface stress sensors need to have a large diameter (several hundred microns) to improve sensitivity.^38^

Optical interference generated in the air gap between the suspended graphene and the substrate can be used to detect nanomechanical deflection associated with molecular adsorption. Adsorbed molecules with equal electric charge produce repulsive coulombic forces that apply a compressive surface stress to the suspended graphene, resulting in upward deflection of the suspended graphene. Meanwhile, the expansion of the nanocavities causes a change in the reflected peak wavelength. Therefore, the presence of adsorbed molecules can be identified by wavelength peak shifts in the optical interferometry signal. In particular, this optical measurement method can evaluate the kinetics of adsorbed biomolecules in solution.

A visible light filter using optical interference between a suspended double-layer graphene and a Si substrate was reported;^39,40^ however, graphene is a transparent material with a transmittance of 97.7%.^41^ Even in single-layer graphene, the optical interference can be obtained from the slight reflection from the suspended graphene, which corresponds to its nanomechanical deflection. The interference peak position is determined by the air gap *d* under the suspended graphene. The reflection spectrum was obtained by numerical calculations using:^42^1
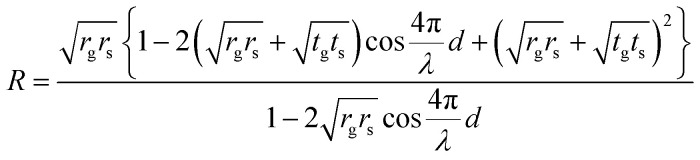
where *R* is the reflectance, *λ* is the wavelength, *t* is the transmittance, and *r* is the reflectance, and the suspended graphene and silicon are denoted by the subscripts g and s, respectively. Therefore, *d* can be evaluated by comparing the experimental and calculated peaks of the reflection spectra.

The reflection spectrum from the graphene-based interferometer was measured using microspectroscopy (ESI Fig. S2†). The graphene drum was blue due to optical interference caused by the slight reflection of the suspended graphene, as shown in Fig. 1(b). Fig. 2 shows the measured reflection spectrum of the graphene interferometer.

**Fig. 2:**
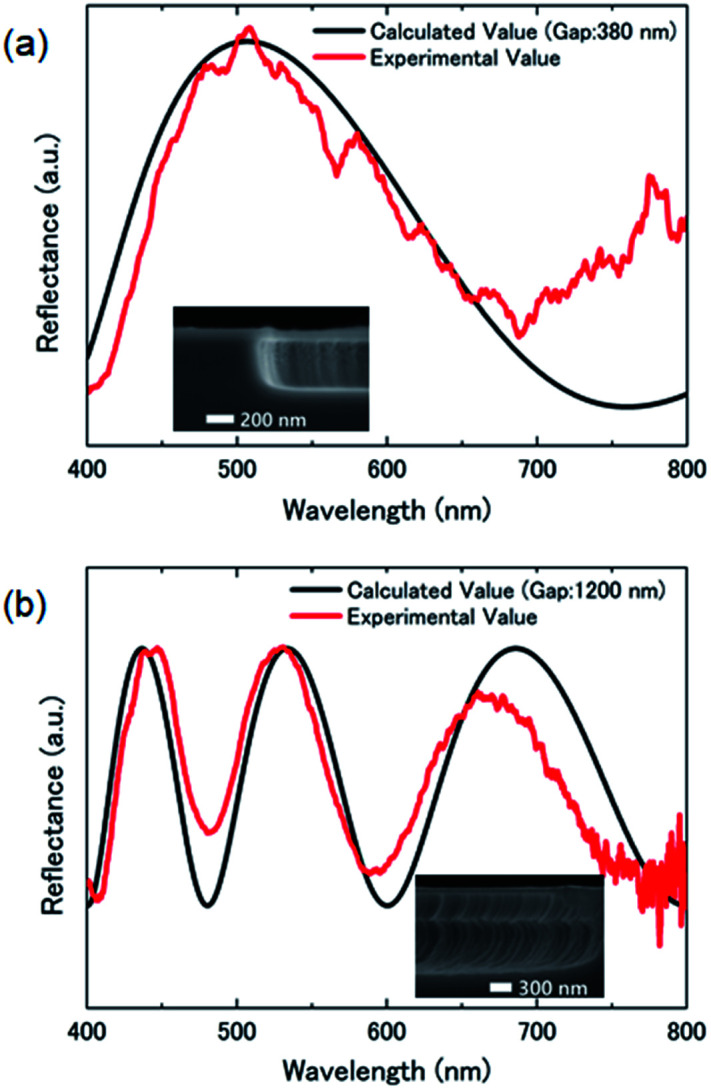
Typical reflection spectrum of the suspended graphene-based optical interferometer with cavity depths of (a) 380 nm and (b) 1200 nm. The solid line is a curve calculated using eqn (1). The cavity gap of the graphene interferometer obtained from the fitting curve was similar to the etching depth formed by RIE during cavity formation.

Cavities of different depths as shown in the inset of SEM images exhibit interference properties depending on the depth. The submicron cavity under the suspended graphene was formed by reactive ion etching (RIE) with CHF_3_ for SiO_2_ etching for 3 min, and using SF_6_ for Si etching for 2.5 min. The total etching depth of 380 nm was as deep as the cavity gap obtained by optical interference calculations using eqn (1). Another interferometer with a 1200 nm deep cavity formed by the deep RIE process created 3 interference peaks in the visible region, which is also in good agreement with the theoretical curve, as shown in Fig. 2(b). Therefore, optical interferometry is an effective method for evaluating the nanomechanical deflection of the suspended graphene associated with selective molecular adsorption.

To demonstrate molecular selectivity using an antigen–antibody reaction, the antibodies need to be immobilized on suspended graphene. Since no dangling bonds exist on the surface of graphene, we used 1-pyrene succinimidyl ester (PBSE) as a cross-linker to immobilize the antibodies.^22,23^ The pyrenyl group of PBSE binds to the graphene surface *via* pi-stacking, while the succinimidyl parts combine with the antibody. Chemical functionalization of PBSE was performed by soaking the graphene chip for 1 h in a solution of PBSE dissolved in deionized water at a concentration of 1 mg mL^−1^. When a chip in a dry state was brought into contact with the PBSE solution, a transient response was observed, where the suspended graphene deformed upward due to the surface tension force of the solution. To avoid this physical influence during chip insertion, the surface tension force of the solution was reduced by adding 1% Tween-20 to the PBSE solution. After functionalization, the graphene chip was rinsed in phosphate buffered saline (PBS) for 5 min. Subsequently, the graphene chip functionalized with PBSE was soaked in anti-bovine serum albumin antibody (anti-BSA) solution at a concentration of 100 μg mL^−1^ for 1 h, followed by immersion in PBS for 5 min. Then, we evaluated the molecular selectivity by immersing graphene chips in BSA antigen solution or human serum albumin (HSA) solution. Note that the surface stress response due to the molecular adsorption depends on the electric charge of the adsorbed molecules.^25,26^ For fair evaluation, it is necessary to select a molecule that has the same molecular weight and isoelectric point as BSA. Although HSA is the same albumin as the BSA antigen, HSA has no cross-activity with anti-BSA antibody (ESI Fig. S3†). Therefore it is not bound to the antibody on the suspended graphene.

Fig. 3(a)–(c) show microscopy images of the suspended graphene at the initial state, after antibody immobilization, and after the antigen–antibody reaction, respectively. The cavity gap *d* between the suspended graphene and Si substrate was 380 nm, which should give a blue color by optical interference at the initial state. The interference color changed to green associated with immobilization of PBSE and anti-BSA on the suspended graphene. It is considered that the suspended graphene was deformed due to immobilization of the molecules, namely the cavity gap increased due to the compressive surface stress, resulting in a red-shift in the interference peak. Furthermore, when the antibody-modified suspended graphene was reacted in 100 ng mL^−1^ BSA antigen solution, the interference color changed to yellow, indicating a further increase in the cavity gap under the suspended graphene. These results clearly prove that the surface stress on the suspended graphene further increased with BSA antigen binding.

**Fig. 3:**
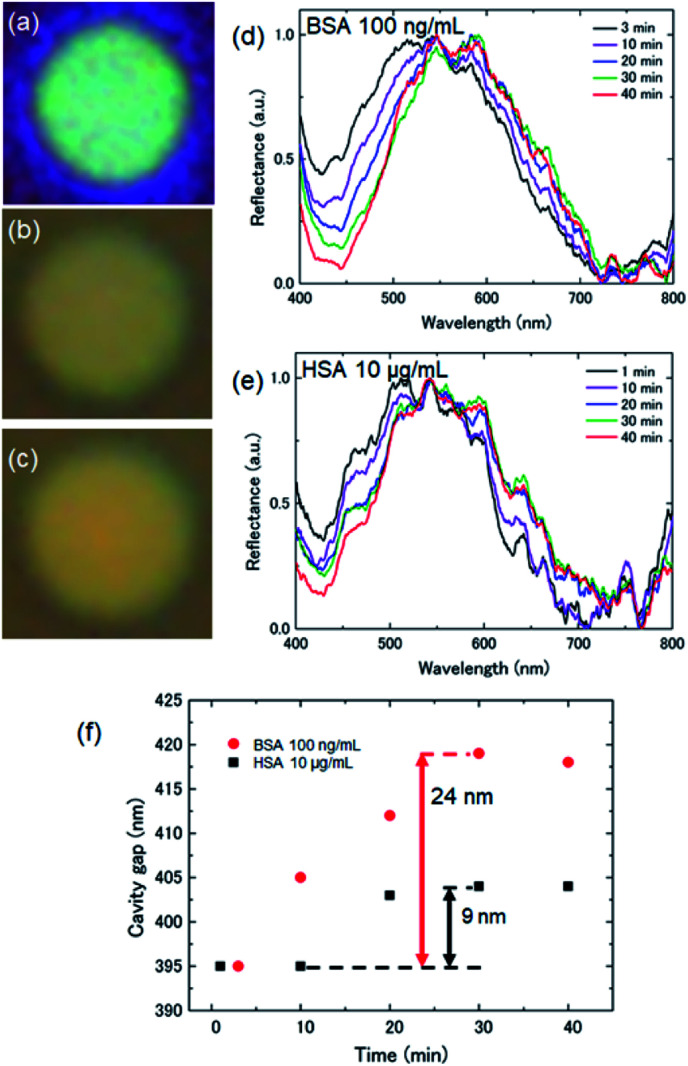
(a–c) Optical microscopy images of the suspended graphene in the initial state, after antibody immobilization, and after the antigen–antibody reaction, respectively. Reflection spectra of the graphene interferometer with a diameter of 6 μm in (d) 100 ng mL^−1^ BSA antigen solution and (e) 10 μg mL^−1^ HSA solution as a function of the reaction time. (f) Time-dependence of graphene deflection associated with binding of molecules.

Fig. 3(d) shows the reflection spectrum of the suspended graphene with a diameter of 6 μm in BSA antigen solution as a function of reaction time. The peak position showed a red-shift of 43 nm from the initial position, indicating that compressive surface stress was applied to the suspended graphene by the bound BSA antigen. In addition, the peak shift saturated after 30 min of reaction, indicating that the suspended graphene stayed in a deformed state. This suggests that the compressive surface stress due to antigen binding and the restoring force of the suspended graphene were in equilibrium.

To demonstrate the selective detection of target molecules on the suspended graphene, reflection spectra were measured by immersing the anti-BSA-conjugated suspended graphene chip in 10 μg mL^−1^ HSA solution, as shown in Fig. 3(e). From the obtained spectral shift, the change in the cavity gap was calculated using eqn (1), as shown in Fig. 3(f). The cavity gap increased by 24 nm and 9 nm in the BSA antigen solution and the HSA solution at saturation, respectively. While the concentration of the HSA solution is 100 times higher than that of the BSA antigen solution, the BSA-treated suspended graphene deformed to a higher degree, attributed to the large number of immobilized molecules. These results suggest that the BSA antigen selectively binds to the suspended graphene surface *via* an antigen–antibody reaction, leading to a deflection double that of suspended graphene with the HSA physically adsorbed. Hence, we successfully demonstrated selective molecular detection by chemical functionalization on cavity-sealed suspended graphene using optical interferometry.

To evaluate the diameter dependence of the membrane response, we performed the same measurements with diameters of 6–10 μm. From the reflection spectra, we determined deflections for diameters of 6, 8, and 10 μm during the antigen–antibody reaction of 39, 70, and 110 nm, respectively, as shown in Fig. 4. The microscopy images show that the reflected color changed from red to yellow with the increasing drum diameter. The color at the center of the 10 μm-diameter drum was red while at it edge it was yellow, indicating convex curvature of the membrane. Curve fitting of the deflection of the suspended graphene was performed using the Stoney formula^36^ as a function of the drum diameter. The measured values were consistent with a quadratic curve, indicating that the nanomechanical deflection at saturation depended on the square of the diameter. It is suggested that the nanomechanical deflection of the suspended graphene depends on the surface stress associated with molecular adsorption.

**Fig. 4:**
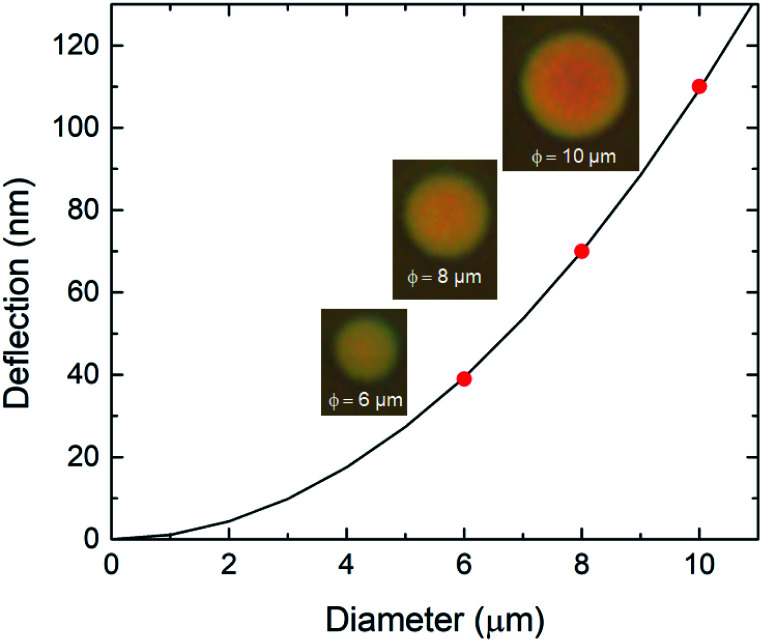
Nanomechanical deflection of the suspended graphene treated with 100 ng mL^−1^ BSA antigen solution as a function of drum diameter. The solid line represents the theoretical curve calculated using the Stoney formula. The insets show microscopy images showing the color change of the graphene interferometers with diameters of 6, 8, and 10 μm after treatment with the BSA antigen.

For the evaluation of concentration dependence, the deflection of a 6 μm-diameter drum to the antigen–antibody reaction was measured with BSA antigen concentrations of 1–1000 ng mL^−1^ (ESI Fig. S4†). From the reflection spectra at saturation, changes in the cavity gap (graphene deflection) were calculated using eqn (1). Fig. 5 shows typical BSA concentration dependence of the graphene deflection. The results indicated that the graphene deflection was dependent on the BSA concentration in this concentration range. At a concentration of 1 ng mL^−1^, no spectral shift was observed before and after the dropping of the BSA antigen solution, indicating that the lower limit of detection was determined to be 10 ng mL^−1^.

**Fig. 5:**
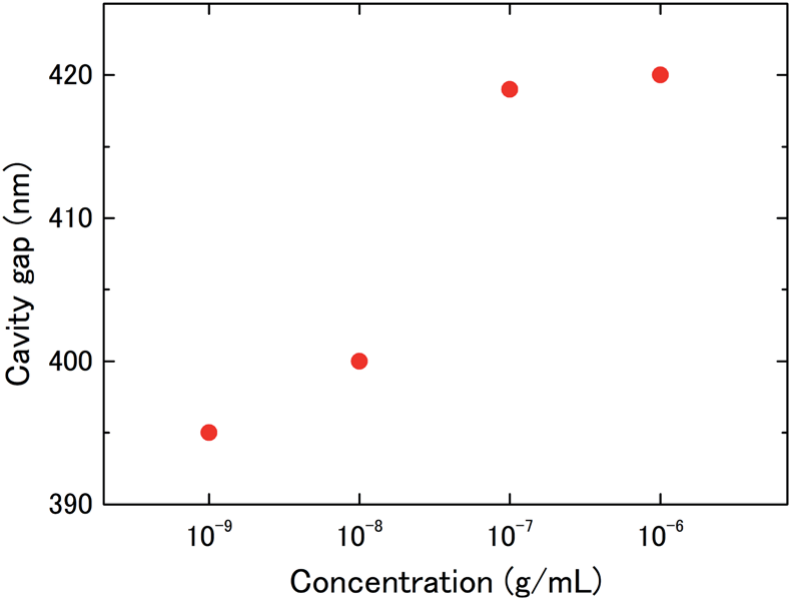
Concentration dependence of nanomechanical deflection of the suspended graphene treated with 1–1000 ng mL^−1^ BSA antigen solution.

In summary, we demonstrated selective molecular detection using a graphene drum, which had sufficient strength to allow wet chemical functionalization. Real-time molecular adsorption in solution was performed by colorimetry using optical interference associated with nanomechanical deflection of the suspended graphene. In addition, the kinetics of the molecular interaction could be evaluated using the suspended graphene based on the detection of surface stresses. Our proposed technique for selective molecular detection achieves highly sensitive chemical sensing and biosensing and offers charge detection, resonant mass detection, and surface stress detection methods. We anticipate that the suspended graphene-based sensor could detect single molecules without labelling. Moreover, the optical interferometric surface stress sensor employs a CMOS image sensor technology for a light receiver and signal processor.^18^ By applying suspended graphene to the optical interferometric sensor, a single sensor pixel including a buffer amplifier can be configured with 10 × 10 μm^2^ or less while the conventional surface stress sensor with the design rule of several hundred microns has no size compatibility with the CMOS image sensor. Therefore, a highly sensitive graphene-based surface stress sensor allows miniaturization and a high-density sensor array for multi-biomarker detection, resulting in an increase to 10^6^ pixels per cm^2^.

## Conflicts of interest

There are no conflicts to declare.

## Supplementary Material

NA-002-C9NA00788A-s001
