# Supplementary material for: A suspended graphene-based optical interferometric surface stress sensor for selective biomolecular detection
Source: Nanoscale Adv. 2020 Mar 19;2(4):1431–6. doi: 10.1039/c9na00788a (PMC9417660; doi:10.1039/c9na00788a)
Supplement: NA-002-C9NA00788A-s001 [file NA-002-C9NA00788A-s001.pdf]

Electronic Supplementary Material (ESI) for Nanoscale Advances.
This journal is © The Royal Society of Chemistry 2020

Electric Supplementary Information

Suspended graphene-based optical interferometric surface stress sensor for selective biomolecular detection

Shin Kidane, Hayato Ishida, Kazuaki Sawada, and Kazuhiro Takahashi*

Department of Electrical and Electronic Information Engineering, Toyohashi University of Technology, 1-1 Hibarigaoka, Tempakucho, Toyohashi, Aichi 441-8580, Japan

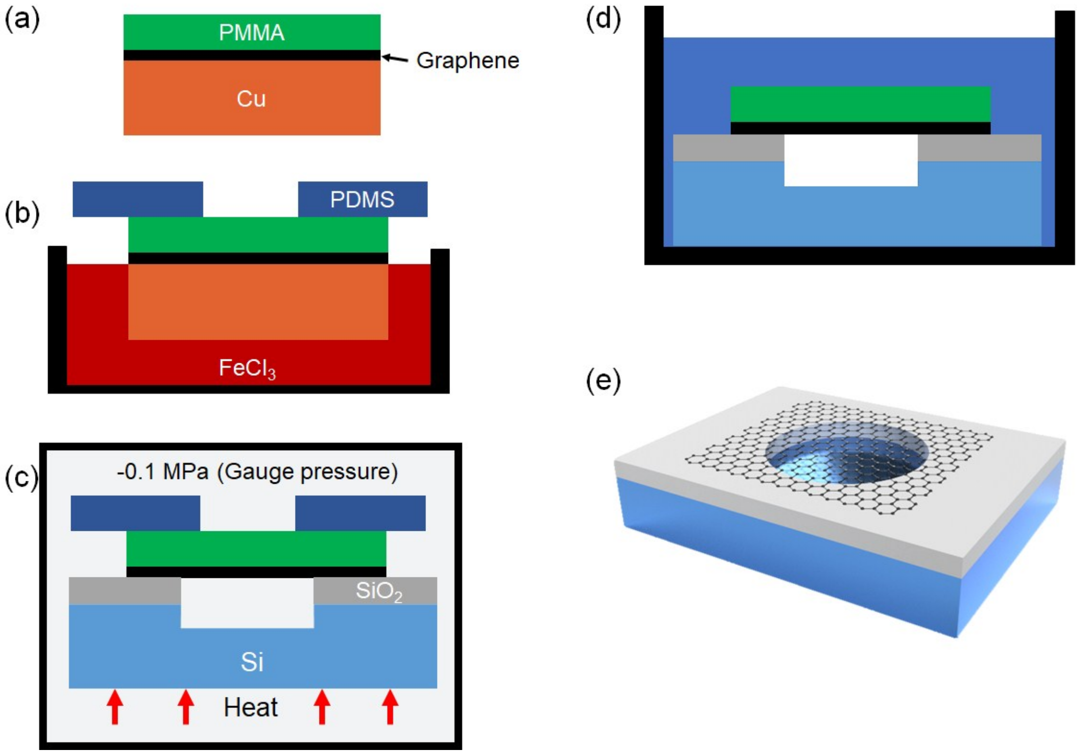

Figure S1. Schematic diagram describing the low-pressure dry-transfer process for depositing CVD-grown graphene onto a Si substrate with pre-patterned nanocavities. Single-layer graphene was deposited by CVD onto Cu foil. (a) A polymethyl methacrylate (PMMA) solution was spin-coated onto the graphene/Cu foil. (b) The Cu layer was removed by etching with an $FeCl_3$ solution while the PMMA/graphene sheet was supported by a polydimethylsiloxane (PDMS) block, followed by rinsing in deionized water. (c) The PDMS/PMMA/graphene was transferred onto a thermal-SiO_2/Si substrate with shallow cavities created by reactive ion etching in a vacuum chamber. The graphene-contacted substrate was heated on a hotplate to increase the temperature above the glass-transition temperature of PMMA, leading to strong adhesion. (d) The supporting PMMA layer was removed using an N-Methyl-2-pyrrolidone-based solvent at 60 ℃. (e) Finally, the suspended graphene was dried by CO_2 supercritical drying.

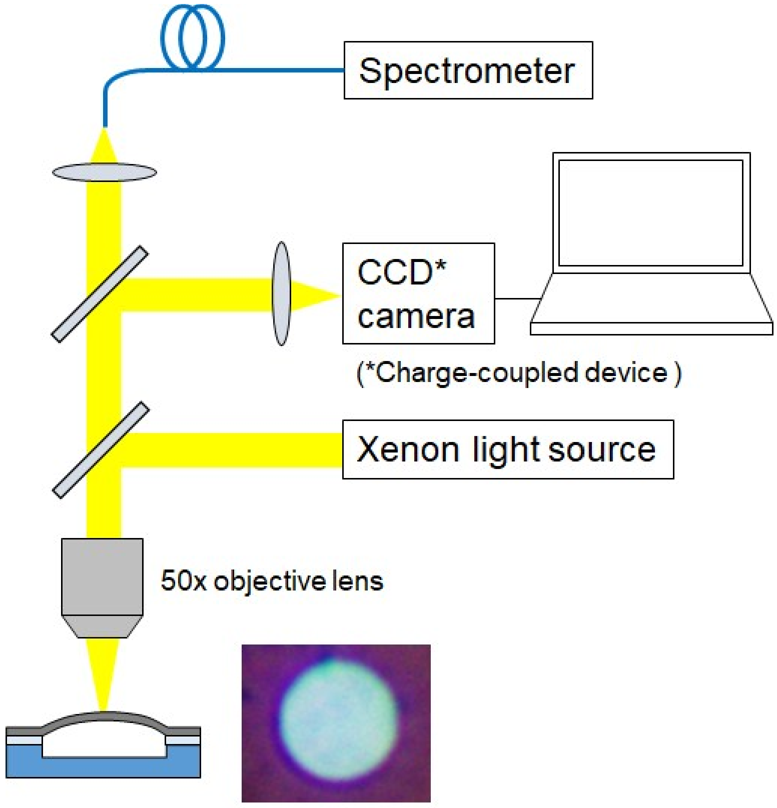

Figure S2. Optical interference measurements for the graphene drums with diameters of ≤10 μm were performed using microspectroscopy. White light from a xenon lamp (Asahi Spectra, LAX-C100) was used to illuminate the sensor chip via a reflection microscope (Olympus, BX51). A visible spectrometer (Ocean Optics, USB2000) was attached to the microscope using an optical fiber with a core diameter of 100 μm. The magnification of the objective lens was 50×, and the spot diameter was 2 μm, which was a sufficiently small measurement area for the fabricated graphene drum.

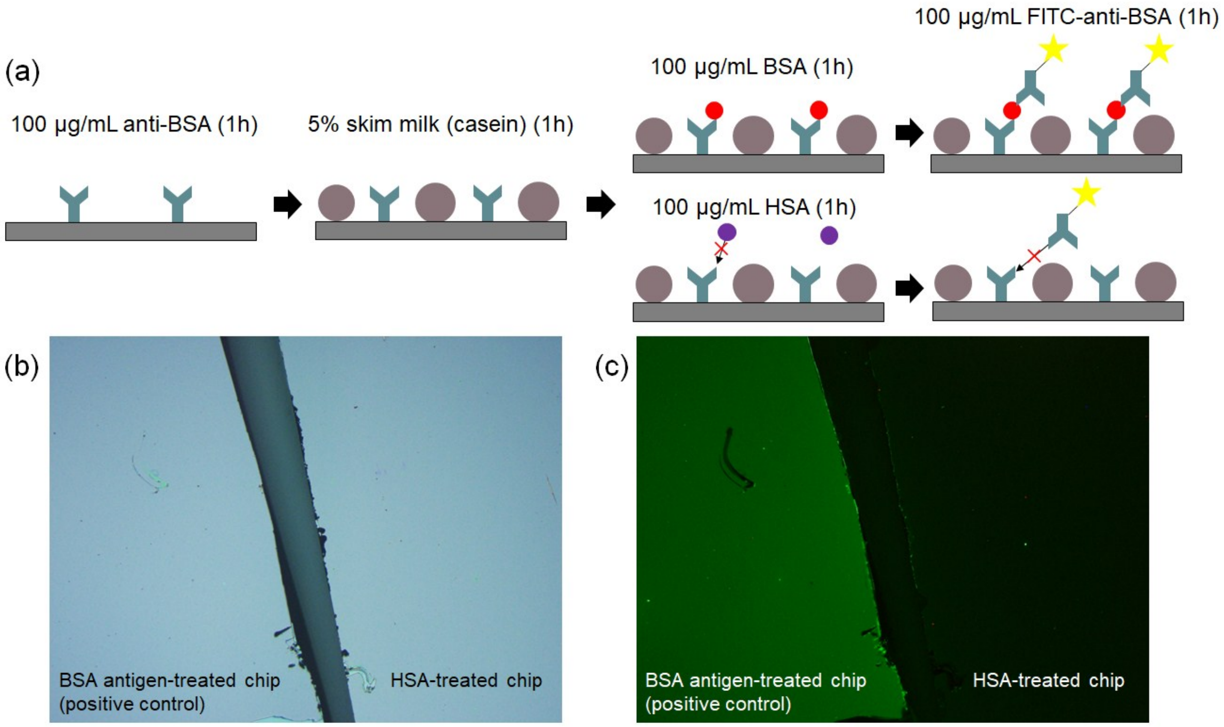

Figure S3. Cross-reactivity between anti-bovine serum albumin (BSA) and human serum albumin (HSA) was evaluated by fluorescence observations. (a) Two types of protocols for treating BSA antigen with anti-BSA antibody (positive control) and treating HSA with anti-BSA antibody are shown. We prepared two substrates modified with anti-BSA antibody (100 μg/mL) and casein for blocking (5% skimmed milk). The anti-BSA-modified chips were immersed in a 100 μg/mL BSA antigen solution and a 100 μg/mL HSA solution, followed by treatment in a 100 μg/mL fluorescein isothiocyanate (FITC)-conjugated anti-BSA antibody solution as a secondary antibody. After each step, the chip was washed with phosphate-buffered saline (PBS). The treated chips were immersed in PBS, and (b) bright field observation and (c) fluorescence observation were performed. Fluorescence was observed only in the BSA antigen-treated chip, indicating that there is no cross-activity between anti-BSA and HSA.

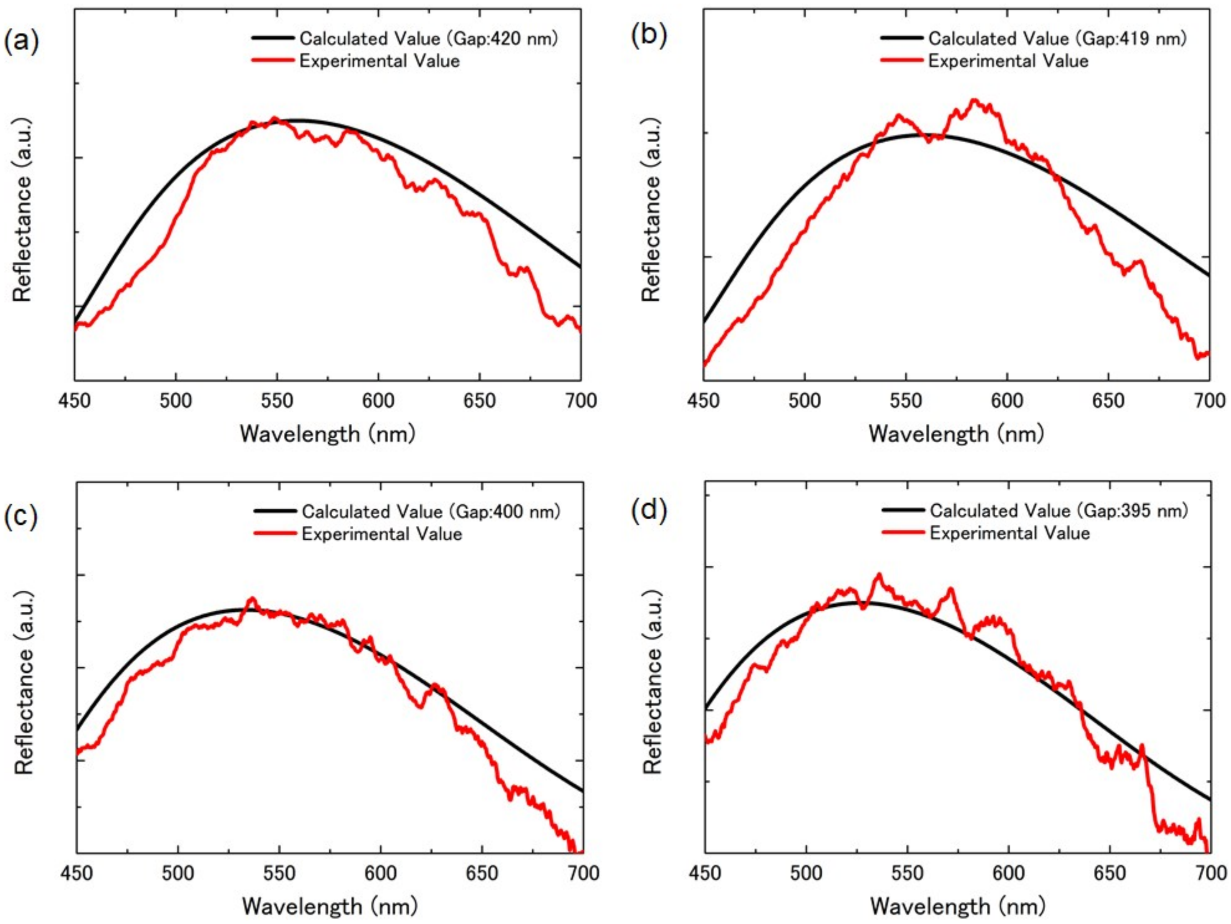

Figure S4. Typical reflection spectra at the saturation of a 6-μm-diameter drum corresponding to BSA concentration; (a) 1 μg/mL, (b) 100 ng/mL, (c) 10 ng/mL, and (d) 1 ng/mL. The fitting curve was drawn using Equation 1 with the air gap as a parameter. The gap change corresponds to the graphene deflection.
